# Actors and Factors in the Resolution of Intestinal Inflammation: Lipid Mediators As a New Approach to Therapy in Inflammatory Bowel Diseases

**DOI:** 10.3389/fimmu.2017.01331

**Published:** 2017-10-23

**Authors:** Federica Ungaro, Federica Rubbino, Silvio Danese, Silvia D’Alessio

**Affiliations:** ^1^Department of Biomedical Sciences, Humanitas University, Rozzano, Italy; ^2^Laboratory of Gastrointestinal Immunopathology, Humanitas Clinical and Research Center, IBD Center, Rozzano, Italy

**Keywords:** resolution of inflammation, pro-resolving lipid mediators, inflammatory bowel disease, polyunsatured fatty acids, pathogenesis, mucosal inflammation, tissue homeostasis

## Abstract

In the last few decades, the pathogenesis of inflammatory bowel disease (IBD) in genetically predisposed subjects susceptible to specific environmental factors has been attributed to disturbance of both the immune and non-immune system and/or to the imbalanced interactions with microbes. However, increasing evidences support the idea that defects in pro-resolving pathways might strongly contribute to IBD onset. The resolution of inflammation is now recognized as a dynamic event coordinated by specialized pro-resolving lipid mediators (LMs), which dampen inflammation-sustaining events, such as angiogenesis, release of pro-inflammatory cytokines, clearance of apoptotic cells, and microorganisms. Among these pro-resolving molecules, those derived from essential polyunsaturated fatty acids (PUFAs) have been shown to induce favorable effects on a plethora of human inflammatory disorders, including IBD. Here, we offer a summary of mechanisms involving both cellular and molecular components of the immune response and underlying the anti-inflammatory and pro-resolving properties of PUFAs and their derivatives in the gut, focusing on both ω-3 and ω-6 LMs. These fatty acids may influence IBD progression by: reducing neutrophil transmigration across the intestinal vasculature and the epithelium, preventing the release of pro-inflammatory cytokines and the up-regulation of adhesion molecules, and finally by promoting the production of other pro-resolving molecules. We also discuss the numerous attempts in using pro-resolving PUFAs to ameliorate intestinal inflammation, both in patients with IBD and mouse models. Although their effects in reducing inflammation is incontestable, results from previous works describing the effects of PUFA administration to prevent or treat IBD are controversial. Therefore, more efforts are needed not only to identify and explain the physiological functions of PUFAs in the gut, but also to unveil novel biosynthetic pathways of these pro-resolving LMs that may be dysregulated in these gut-related disorders. We suppose that either PUFAs or new medications specifically promoting resolution-regulating mediators and pathways will be much better tolerated by patients with IBD, with the advantage of avoiding immune suppression.

## Introduction

Inflammatory bowel diseases (IBDs), encompassing ulcerative colitis (UC), and Crohn’s disease (CD) are immunologically mediated inflammatory disorders of the gut, whose prevalence and incidence are dramatically increasing worldwide. Although clinical manifestations of these diseases are different, they share common features. In fact, both UC and CD are characterized by epithelial barrier damage that allows commensal bacteria and microbial products to translocate into and colonize the intestinal wall. This event triggers the release of cytokines, chemokines, and eicosanoids which thanks to regulatory mechanisms, mediate the physiological self-limiting immune-response (1, 2). Furthermore, both immune and non-immune components of the intestinal mucosa have been shown to exert a key role in IBD pathogenesis (3). In terms of immune components, the innate and the adaptive immune system are essential to chronic intestinal inflammation. In fact, innate immune cells (e.g., neutrophils, monocytes, and macrophages) hold the capability to remodel the response of adaptive T cells during the inflammatory process (4). Concomitantly, studies of the intestinal microbiota, environmental factors, and genetics have identified a significant contribution of non-immune components to the pathogenesis of IBD, which include: breach in the epithelial wall, that is, the first line of gut defense against bacteria and other microorganisms (5–7); defects in the biological activities of stromal cells, which hold immune-modulatory actions and the capability to clear chemokines and cytokines from the inflammatory milieu to re-establish mucosal homeostasis (8, 9); defective endothelial cell functions, crucial for the angiogenic process but also for the regulation of leukocyte adhesion, and trafficking across the hematic and lymphatic barriers (10–14). Activities of both immune and non-immune cells need to be finely modulated and constantly balanced, in order to avoid chronicity of inflammation and tissue damage.

Another key component of IBD pathogenesis is represented by the gut microbiota (15). In fact, the gastrointestinal tract hosts the largest microbial community of the organism that can be shaped by environmental factors, diet, and hygiene during childhood (16), whereas in adulthood this is more stable with a defined composition of bacteria (17, 18). In healthy subjects, homeostasis exists between the intestinal microbiome, mucosal barrier, and immune system. In IBD, this homeostasis is altered causing a “dysbiosis,” disrupted barrier function as well as immune system activation (15).

Although many efforts have been made to delineate the causes underlying the exact etiopathogenesis of IBD, so far our knowledge does not fully clarify what causes its onset. It is currently well accepted that at the basis of IBD pathogenesis (19, 20) there is an imbalance between pro- and anti-inflammatory signals (1). This suggests that defects in the proper release of pro-resolving molecules during the acute phase of inflammation may characterize IBD onset. For decades, the resolution of inflammation has been considered a passive event, in which pro-inflammatory signals simply dilute over time. This concept was overturned when Serhan and colleagues discovered for the first time that a specific class of lipids, known as eicosanoids and docosanoids, promotes, and orchestrates the resolution process (21, 22).

This discovery gave rise to a new field of research studying the mechanisms and the factors involved in the resolution phase of the inflammatory response, which is finely and temporally regulated by specialized pro-resolving mediators, named lipoxins (LXs), resolvins (Rv), protectins, and maresins. These resolving bioactive lipids are synthesized from ω-6 and ω-3 polyunsaturated fatty acids (PUFAs) and have been demonstrated to exert potent immune-resolving effects (2). However, this line of research is still at its infancy in the IBD field.

In fact, the vast majority of therapies currently in use for IBD aims at blocking key inflammatory mediators that are triggered during the early stages of acute inflammation. However, targeting infiltrating immune cells does not always lead to remission or stable resolution. Indeed, conventional anti-inflammatory agents do not alter the course of IBD because the naturally occurring resolution programs are likely to be subverted. For this reason, promotion and maintenance of the resolving milieu may represent a good alternative therapeutic approach to dampen chronic inflammation in IBD. In addition, defects in the production of resolving molecules may strongly contribute to IBD onset, thus expanding our understanding of what triggers these gut-related diseases.

This review aims to describe how the resolution process plays a fundamental role in the gut both at the physiological and pathological level. After a brief overview on IBD pathogenesis, we will emphasize which cellular and molecular components govern the resolving phase of intestinal inflammation and we will discuss the state of the art of preclinical and clinical studies employing PUFA-derived pro-resolving lipid mediators (LMs) in IBD.

## Resolution of Inflammation and Pro-Resolving LMs: A Brief Overview

For decades, anti-inflammatory treatments have been used to treat chronic inflammatory conditions because of the concept that the chronically established inflammation was caused by an exaggerated immune response rather than a failure in the resolution of inflammation (23). Indeed, for years the resolution process has been considered a passive event where inflammatory signals progressively dissipate (2, 24). In contrast to this assumption, during the last decade resolution of inflammation has been conclusively recognized as an active and tightly regulated process orchestrated by pro-resolving LMs, which have been found to dampen inflammation-sustaining events such as cell proliferation, migration, and clearance of apoptotic cells and microorganisms (2, 25).

At tissue and cellular level, the key steps that characterize the resolution process are (i) clearance of the inciting stimuli, (ii) silencing of pro-inflammatory and local survival signals, including chemokine gradients, (iii) polymorphonuclear (PMN) efferocytosis and clearance by tissue and monocyte-derived macrophages, and (v) recirculation of macrophages *via* lymph flow. LMs represent the key signaling molecules in this process, which regulate the inflammatory profile and promote the return of affected tissues to homeostasis (26).

In this context, ω-3 and ω-6 PUFAs are specialized LMs that have the capability of influencing the inflammatory processes, such as those governing IBD. They are essential fatty acids that need to be obtained from the diet; in fact, since mammals lack of endogenous enzymes necessary for ω-3 PUFA desaturation, they cannot be synthesized by humans (27).

**Figure 1:**
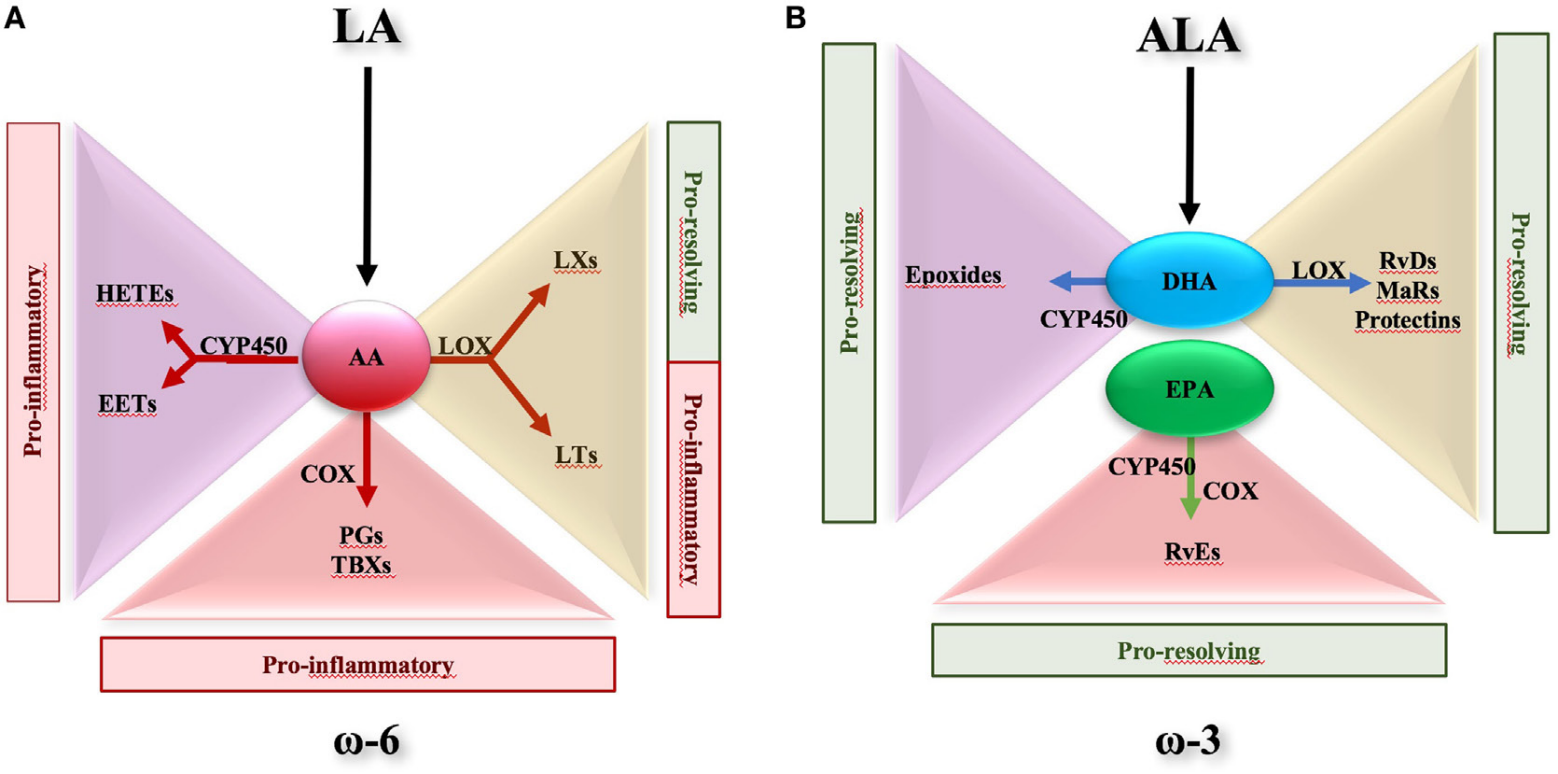
Metabolic route of ω-6- and ω-3-derived lipid mediators. **(A)** Essential fatty acid linoleic acid, classified as ω-6 polyunsaturated fatty acid, can be converted into arachidonic acid (AA). In turn, AA is metabolized in hydroxy-eicosatetraenoic acids (HETEs) and epoxy-eicosatrienoic acids (EETs) *via* cytochrome P450 (CYP450). *Via* lipoxygenase (LOX) pathway, AA is converted to lipoxins (LXs) and leukotriens (LTs), whereas *via* cyclooxygenase it is metabolized in prostaglandins (PGs) and tromboxanes (TBXs). HETEs, EETs, PGs, TBXs, and LTs are all pro-inflammatory, while LXs are considered pro-resolving mediators. **(B)** Essential fatty acid α-linolenic acid is converted to eicosapentaenoic acid (EPA) and docosahexaenoic acid (DHA). EPA and DHA may be substrate of CYP450, resulting into production of E-series resolvins (Rv) and epoxides, respectively. In addition, DHA is metabolized *via* LOX to D-series Rv, maresins, and protectins. All these EPA- and DHA-derived mediators are recognized to harbor pro-resolving properties.

Polyunsaturated fatty acid metabolism is recognized as an important factor in immune regulation and disease control. In particular, the metabolic balance between ω-6 and ω-3 PUFAs is widely held to be important in human health and diseases (27–30). PUFA-derived bioactive metabolites are formed *in vivo* by enzymatic oxidation through the action of cyclooxygenases (COXs), lipoxygenases (LOXs), and cytochrome P450 (CYP450) monooxygenases. From ω-6 PUFAs, e.g., arachidonic acid (AA), the COX pathway leads to the formation of prostanoids, such as prostaglandins (PGs) and thromboxanes (TXs), the LOX pathway leads to leukotrienes (LTs) and LXs, and the CYP450 pathway gives rise to hydroxy-eicosatetraenoic acids (HETEs) and epoxy-eicosatrienoic acids (Figure 1A) (2, 24, 31, 32). Except for LXs (33), ω-6 PUFAs are conventionally involved in the initiation of inflammatory responses. On the contrary, ω-3 PUFAs seem to promote resolution of inflammation (34). α-linolenic acid (ALA) is an ω-3 PUFA and is categorized with the ω-6 linoleic acid (LA) as an essential fatty acid. As ω-6 LA can be metabolized into AA, ALA can be converted into precursors for long chain ω-3 PUFAs such as eicosapentaenoic acid (EPA) and docosahexaenoic acid (DHA). Both EPA and DHA, which can be found in some fish oils, are good substrates for LOX and CYP, thus being efficiently converted into bioactive metabolites such as E-series resolvins (RvEs), D-series resolvins (RvDs), protectins, and maresins that act as potent pro-resolving endogenous mediators in a wide range of human inflammatory disorders, including IBD (Figure 1B) (35–44). A large number of studies sustain the anti-inflammatory potential of EPA and DHA and their derivatives [for a recent review, see Ref. (35, 39)]. Nevertheless, the molecular mechanisms by which these essential fatty acids exert their anti-inflammatory effects remain controversial, particularly in the gut.

Inflammatory bowel disease patients may display a deficiency in essential fatty acids and/or a defect in PUFA biosynthesis and metabolism. This is why the intake of ω-3 PUFAs may benefit patients with both UC and CD by a series of beneficial events such as inhibition of natural cytotoxicity, and improvement of oxidative stress (35, 45–47). This concept is strengthened by the fact that the intestinal mucosa seems to be highly responsive to ω-3 long-chain PUFAs (47–49).

## Actions of Pro-Resolving PUFAs and Target Cell Types in the Gut

Active resolution of inflammation is characterized by a sequential series of events that starts from building an adequate inflammatory response against inciting agents, to minimizing local tissue damage. In this context, pro-resolving PUFAs act with various signals and mechanisms to different cell compartments, with the final purpose to remodel and clear healed tissues of unnecessary immune cells, thus bringing the inflamed organ to the original homeostasis.

The intestinal epithelium is a key coordinator of both inflammation and resolution. Thanks to tight junctions (TJs), intestinal epithelial cells form a dynamic barrier protected by a thick mucus layer which controls what can reach the lamina propria from the lumen (50, 51). In IBD pathogenesis, altered intestinal barrier functions, in terms of decreased mucous production (52) and reduced expression of TJs (53), have been associated with increased gut permeability, which facilitates the absorption of microbial products and triggers an excessive response, eventually leading to mucosa injury in both CD and UC (54, 55). In order to counteract pathogen infections, epithelial cells are able to produce and release in the luminal mucus antibacterial and endotoxin-neutralizing molecules called bactericidal permeability-increasing protein (BPI). BPI damages the membranes of Gram-negative bacteria, neutralizes endotoxin, and opsonizes bacteria for neutrophil phagocytosis (56). BPI is transcriptionally up-regulated by LXs and resolvin E1 (RvE1) (57). In addition, it was observed that RvE1 significantly upregulates the expression of intestinal alkaline phosphatase (ALPI), an enzyme whose activity is critical for the maintenance of bacterial homeostasis (57): for its luminal location, ALPI has been demonstrated to block Gram-negative growth such as *Escherichia coli* and strongly neutralizes LPS through dephosphorylation of moiety in lipid A (58). This was confirmed in the mouse model of dextran sodium sulfate (DSS)-induced colitis, during which the *in vivo* induction of ALPI by RvE1 positively correlated with the resolution process (57).

Lipoxins have also demonstrated to exert an *ex vivo* cytoprotective role on intestinal epithelial cells (59). Goh and colleagues showed that administration of LXs significantly ameliorates TNF-α-induced mucosal inflammation and reduces epithelial cell apoptosis. However, the mechanisms through which LXs exert these cytoprotective effects remain yet to be defined (33).

**Figure 2:**
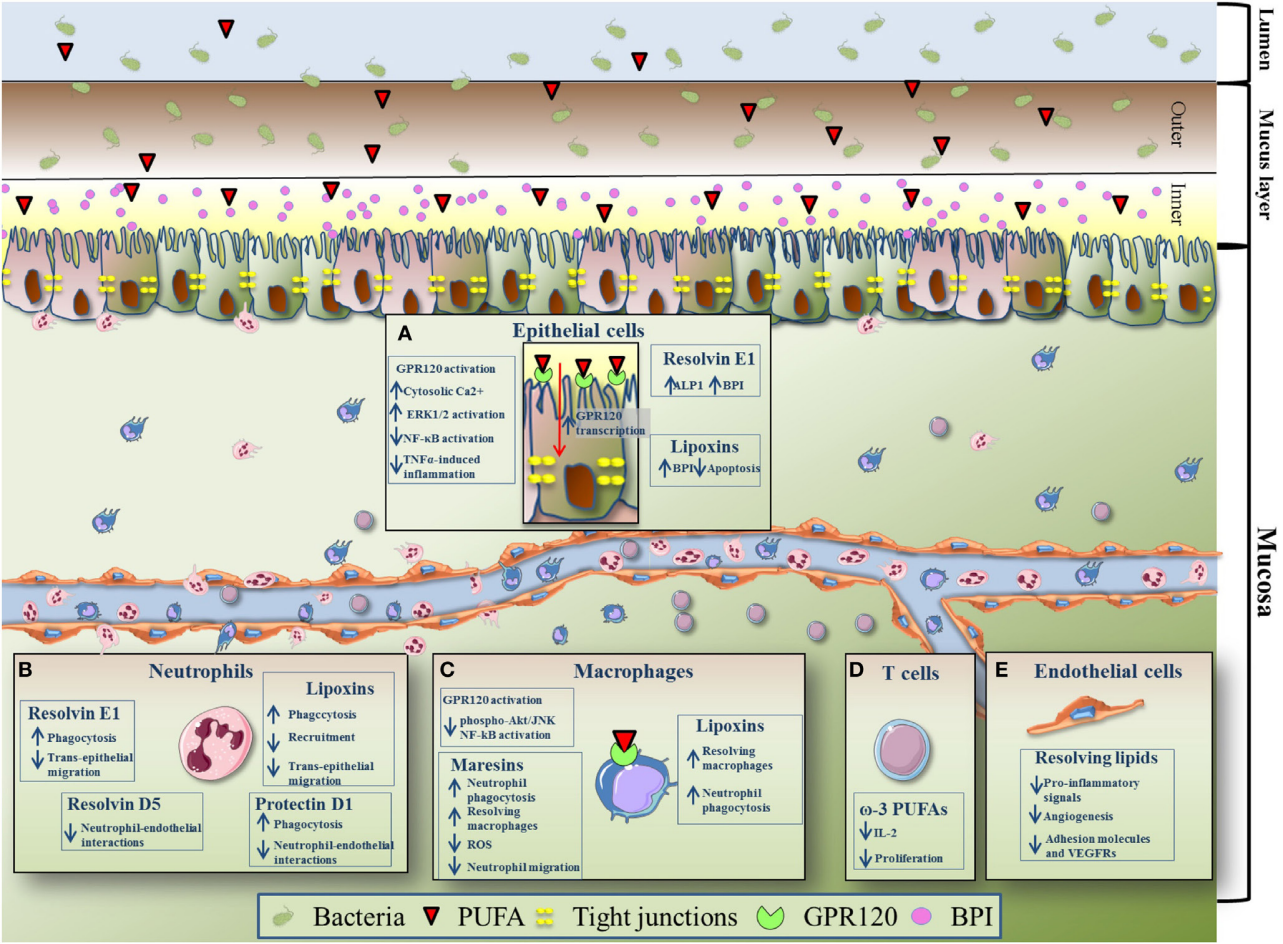
Effects of pro-resolving polyunsaturated fatty acids (PUFAs) on immune and non-immune intestinal components. **(A)** Thanks to tight junctions, intestinal epithelial cells form a dynamic barrier protected by a thick mucus layer (inner and outer) which controls what can reach the lamina propria from the lumen. In order to counteract pathogen infections, epithelial cells are able to produce and release in the luminal mucus antibacterial and endotoxin-neutralizing molecules called bactericidal permeability-increasing protein (BPI). BPI is transcriptionally up-regulated by lipoxins (LXs) and resolvin (Rv) E1. In addition, it was observed that resolvin E1 (RvE1) significantly upregulates the expression of intestinal alkaline phosphatase. Moreover, LXs inhibit epithelial cells apoptosis. G protein-coupled receptor (GPR)120 activation by PUFAs [eicosapentaenoic acid (EPA), docosahexaenoic acid (DHA), and arachidonic acid] leads to accumulation of cytosolic Ca^2+^, activation of MAP kinase ERK1/2, inhibition of IL-1β-induced NF-κB activation, and TNFα-induced inflammation. Transcription of *GPR120* is increased by bacteria belonging to the bacteroides, proteobacteria, and firmicutes phyla. **(B)** Neutrophils (polymorphonuclear) are the first immune cells recruited to the site of inflammation, but are also important players in the first stages of the resolution program. LXs reduce neutrophil recruitment to the inflamed tissue, transepithelial migration, and phagocytosis. Protectin D1 promotes neutrophil phagocytosis. Similar to LXs, RvE1 reduces neutrophil transepithelial migration and induces neutrophil phagocytosis. Moreover, both protectin D1 and RvD5 have been shown to reduce neutrophil–endothelial interaction. **(C)** Macrophages, important for the resolution of intestinal inflammation, express high level of GPR120. EPA- and DHA-dependent activation of GPR120 has been shown to repress Akt/JNK phosphorylation and NF-kB induction. LXs enhance non-phlogistic phagocytosis of apoptotic neutrophils by macrophages. Treatment with LXs may also polarize intestinal macrophages into a resolving phenotype, thus promoting resolution of inflammation. Maresins exert potent pro-resolution and anti-inflammatory activities, ultimately leading to reduced neutrophil migration and increase macrophage phagocytic activities. Maresins induces also the resolving phenotype of macrophages and inhibit reactive oxygen species production. **(D)** EPA and DHA (ω-3 PUFAs) inhibit T cell proliferation and reduce IL-2 production. **(E)** Pro-resolving lipid mediators (DHA, α-linolenic acid-derived) exert anti-inflammatory and anti-angiogenic effects on the gut endothelium. They reduce the production of IL-6, IL-8, GM-CSF PGE-2, and LTB-4 (pro-inflammatory signals), decrease the levels of adhesion molecules (intercellular adhesion molecule 1 and vascular cell adhesion protein 1), and vascular endothelial growth factor receptor 2, thus suppressing the angiogenic component of inflammation.

Polyunsaturated fatty acids have been shown to modulate other biological activities of intestinal epithelial cells. It is known that pro-resolving LMs exert their functions by binding with cell surface receptors, the majority of which belongs to G protein-coupled receptors (GPRs) (60). Among these receptors, GPR120 has been found to be the most abundantly expressed in the gut, particularly on epithelial cells and macrophages (59, 60). A study from Mobraten and colleagues shows that DHA, EPA, or AA are able to trigger GPR120 in Caco-2 cells, initiating multiple and independent signaling processes with different kinetics and intensity; these are (i) the activation of MAP kinases, (ii) the inhibition of IL-1β induced NF-κB activation, and (iii) the cytosolic accumulation of Ca^2+^ (61). Another group shows differential effects of activation of GPR120 by DHA in human intestinal Caco-2 and murine STC-1 cells, two different cell lines representing the mammalian intestinal epithelial layer. In this study, GPR120 stimulation by ω-3 PUFAs increased β-arrestin2 interaction with TAB 1 and attenuated TNFα-induced inflammatory effects by association of TAB 1 with TAK1, which resulted in reduced activation of NF-kB (59). Anti-inflammatory effects exerted by PUFAs through GPR120 were confirmed *in vivo* by Zhao et al., who demonstrated that triggering of GPR120 by DHA treatment ameliorate the experimental colitis in IL-10 deficient mice (62). Interestingly, transcription of *GPR120* in intestinal epithelial cells was found tremendously increased by bacteria belonging to the *Firmicutes, Bacteroides and Proteobacteria phyla* (63), all classified as microorganisms harboring anti-inflammatory properties. This is intriguing, because the dysbiosis observed in patients with IBD is basically caused by a diminished diversity of *Firmicutes* (64). This suggests that reduced expression levels of GPR120 may be one of the causes underlining IBD pathogenesis, and that targeting this receptor may represent a new therapeutic strategy in IBD; however, to date there are no studies that deeply characterize and quantify GPR120 in the inflamed mucosa of IBD patients and further studies to elucidate this aspect are needed. The effects of PUFAs on intestinal epithelial cells are schematically summarized in Figure 2A.

Neutrophils (PMN leukocyte) are the first cell type of the innate immune system to reach inflamed areas and hold the essential role of limiting the invasion of microorganisms (65). In fact, upon transmigration through activated endothelial cells, PMNs infiltrate the intestinal epithelium, and once reached the apical portion of epithelial cells, they come into contact with tons of bacterial stimuli, which further sustain PMN activation. PMN accumulation within the intestinal crypts has been associated with transepithelial resistance (66, 67) and epithelial barrier integrity (68), and in IBD the persistent and prolonged PMN flux across the epithelium has been shown to cause mucosal ulceration and barrier disruption, ultimately facilitating microorganism entry into the submucosa (69), and contributing to the clinical syndrome of malabsorption and diarrhea in these patients (31, 68). However, PMNs are also recognized as important players in the first stages of the resolution program. For example, they release pro-inflammatory LMs (e.g., PGI2 and LTB4) during early inflammation, before producing pro-resolving molecules, such as LXs, Rvs, and protectins at the onset of resolution (21). Due to this dual role, PMN activity needs to be finely regulated in order to reduce tissue damage and avoid chronicity of diseases (70, 71). LXs deriving from the metabolic route of AA, have been shown to inhibit PMN flux across the epithelial barrier (21, 72). In line with this, patients with severe UC display colonic deficiency in LX biosynthesis, which causes low to absent production of lipoxin A4 (LXA4) (73). Accordingly, LXA4 analogs dampen colitis induced by 2,4,6-trinitrobenzene sulfonic acid (TNBS) or DSS (74, 75). RvE1 has been also shown to inhibit PMN transepithelial migration, and TNBS-induced colon damage (Figure 2B) (36, 57). These LMs, that include protectin D1, not only also support phagocytosis of apoptotic PMNs (76), but also mediate the overexpression of C–C chemokine receptor type 5 receptors on apoptotic neutrophils, thus sequestering inflammatory chemokines such as chemokine (C–C motif) ligand 3 and CCL5, and promoting PMN clearance at sites of inflammation (77).

During intestinal inflammation, PMNs not only represent the target cell type of many pro-resolving PUFAs, but they are also the main producers of many molecules. In fact, a number of recent studies (78–81) clearly indicate that activated PMNs generate crucial anti-inflammatory and pro-resolving mediators that characterize the onset of resolution (82, 83). This aspect has been convincingly demonstrated *in vivo*, by depletion of circulating PMNs with anti-Gr1 antibodies, which resulted in the exacerbation of colitis in various mouse models of IBD, implicating PMNs as a key protective factor in ongoing intestinal inflammation (84). This may justify the controversial role exerted by neutrophils to the pathogenesis of IBD, and why their contribution may differ between CD and UC (85). In fact, while in patients with active UC it has been observed a correlation between the extent of PMN infiltration and the severity of the disease (86), several other studies have reported PMN dysfunction in patients with CD (87–89).

Resident macrophages, located in the sub-epithelial layers of the gut, are designated for protecting the host against pathogens and for regulating mucosal responses to commensal bacteria. For this reason, they are considered important players in the resolution of inflammation (90). These cells of the innate immune system have the characteristic to express various GPRs, including GPR120 (19, 91). EPA- and DHA-dependent activation of GPR120 has been shown to have anti-inflammatory activities in both RAW 264.7 monocytes and primary intraperitoneal macrophages; these effects were abolished by GPR120 silencing (92). In another study, PUFA-dependent signaling cascade that follows GPR120 activation in the gut was observed also in macrophages, where the stimulation of this receptor led to the repression of Akt/JNK phosphorylation and NF-kB-mediated cyclooxygenase-2 (COX-2) induction (92–95). Blood-derived macrophages, that in chronic IBD are known to secrete inflammatory cytokines and tissue-degrading proteases (96, 97), and that well differentiate from resident macrophages, are recognized as either perpetuator of inflammation or effectors of the resolution process (33). Treatment of monocyte-derived macrophages with LXA4 and its analogs induced a strong enhancement in phagocytosis of apoptotic neutrophils (98), thus attributing to these PUFA derivatives an additional role in the resolution of intestinal inflammation.

Following studies on macrophages during the resolution process, a new pathway capable of producing potent mediators from DHA has been uncovered and the resulting metabolites have been coined maresins (MaR1 and MaR2), which exert potent pro-resolution and anti-inflammatory activities, ultimately leading to reduced neutrophil migration and increased macrophage phagoytic activities (99–102). Marcon and colleagues recently showed that MaR1 may cause a switch in the macrophage phenotype from the pro-inflammatory “classically activated M1” to the pro-resolving “alternatively activated M2,” as well as direct blockade of PMN transmigration and reactive oxygen species production, which could explain, at least in part, the beneficial actions of this LM in experimental colitis (103). The effects of PUFAs on macrophages are schematically summarized in Figure 2C.

Studies on the effects of PUFA derivatives on the adaptive immune system in the gut are still in their infancy. In general, both DHA and EPA were observed to reduce *in vitro* T cell proliferation and to decrease the expression of both Th1 and Th2 cytokine IL-2 (Figure 2D). Recent works have also unveiled the effects of ω-3 PUFAs on Th17 cells (104–106). However, only few *in vivo* studies have shown a real effect of pro-resolving LMs in T cell reactivity in the gut; these will be described in the paragraph on animal studies.

The excessive transfer of various immune cell types from the peripheral blood to the affected gastrointestinal tracts of IBD patients, depends not only on surface molecules expressed by activated leukocytes, but also on high levels of adhesion molecules expressed by endothelial cells (14). Endothelial cells are key regulators of the inflammatory response, not only providing in the steady state an anti-inflammatory and anti-coagulatory surface, but also controlling which cell type enters the site of inflammation (107). Thus, alterations of endothelial cells may cause an imbalance between initiation of pro-inflammatory mechanisms and those that promote resolution and restitution of tissue homeostasis, ultimately leading to chronic inflammatory disorders, such as IBD. Patients with IBD are indeed characterized by increased vascularization, and excessive release of angiogenic factors (108, 109). Resolving LMs were observed to exert anti-inflammatory and anti-angiogenic effects on the gut endothelium. For example, Ibrahim and colleagues demonstrated that DHA is able to decrease vascular cell adhesion protein 1 (VCAM-1), TLR4, COX-2, and vascular endothelial growth factor receptor 2 (VEGFR-2) expression and reduce the production of IL-6, IL-8, GM-CSF, and PGE-2 in intestinal microvascular endothelial cells (HIMEC) stimulated with IL-1β. Moreover, administration of ALA during the TNBS model resulted in the decrease of intercellular adhesion molecule 1 (ICAM-1), VCAM-1, and VEGFR-2 expression levels, thus leading to suppression of angiogenesis in the inflamed colon (Figure 2E) (110). Interestingly, Ungaro et al. demonstrated that the Major Facilitator Superfamily Domain containing 2A (MFSD2A) may act as a new player in the resolution of intestinal inflammation, likely promoting endothelial production of DHA-derived pro-resolving mediators (20). In this study, lentiviral induction of MFSD2A conferred anti-angiogenic properties to HIMEC, reducing *in vitro* capillary formation and proliferation, and significantly inhibited TNFα-triggered inflammatory machinery of NF-kB signaling, *via* production of pro-resolving DHA-derived metabolites. These findings suggest that stimulating MFSD2A activity in intestinal endothelial cells could be a novel and powerful therapeutic approach to treat IBD.

Overall we have reported that the main modes of action of PUFAs in the inflamed gut are: (i) inhibition of leukocyte chemotaxis, reduced expression of adhesion molecules, and diminished leukocyte-endothelial adhesive interactions, (ii) modulation of epithelial biological functions and interactions with PMN, (iii) suppression of macrophage phagocytic activities, (iv) production of inflammatory cytokines, and (v) modulation of endothelial functions and T-lymphocyte reactivity. However, there are other mechanisms of action that have not been described in the intestine, but that may be crucial for further studies in IBD. For example, it has been demonstrated that resolving ω-3 PUFAs, such as EPA and DHA, can compete with the enzymes that convert AA into pro-inflammatory eicosanoids, thus inhibiting the release of inflammatory cytokines (e.g., TNF-α and IL1-β) (111). Furthermore, administration of ω-3 PUFA derivatives may benefit IBD patients by change in the lipid composition of intestinal cell membranes, activation of anti-inflammatory proteins such as the transcription peroxisome proliferator activated receptor γ (PPAR-γ), and reduction in the gut production of pro-inflammatory molecules, like NF-κB, LTs, and PGs (35, 45–47, 112, 113).

## Role of PUFAs in Animal Models of IBD

One of the first studies unveiling the contribution of PUFAs in IBD progression was done by Hudert et al., who exploited a transgenic mouse carrying *Caenorhabditis elegans fat-1* gene, encoding for a specific desaturase capable of producing ω-3 PUFAs from ω-6 PUFAs (114). As a consequence, this transgenic mouse is characterized by a low ratio of ω-6/ω-3 fatty acids in its tissues and organs (115). They showed that *fat-1* transgenic mice subjected to the DSS protocol of chemically induced experimental colitis, had significantly reduced signs of colon inflammation, in terms of both clinical manifestation and pathology, than wild-type littermates. Such amelioration was positively correlated with the production of anti-inflammatory ω-3 PUFA derivatives, reduced levels of pro-inflammatory cytokines and a concomitant increase of mucus-specific factors in their colons. Moreover, beside a reduced number of Th17 cells in lymphoid tissues, they also observed a reduced expression of Th17 cell type-specific cytokines and chemokine receptors specifically in the colonic mucosa, indicating a role for ω-3 PUFAs on T cell reactivity. The reduced susceptibility to chemically induced colitis in *fat-1* mice is likely to result from reduced activation of the NF-κB pathway and decreased expression of TNFα, IL-1β, and inducible NO synthase. Conversely, the enhanced protection conferred by a thicker mucus layer in these mice was probably due to the concomitant up-regulation of trefoil factor 3, toll-interacting protein, and zonula occludens-1.

Initial studies on the efficacy of PUFAs in animal models of IBD considered the use of PUFA precursors instead of single metabolites. One of the first fatty acid used in experimental colitis was conjugated LA (CLA), a mixture of 28 isomers of LA (116); this has been tested in pig models of colitis. Animal treated with CLA showed reduced signs of intestinal inflammation, accompanied by decreased serum levels of TNF-α and NF-κB, and increased amount of transforming growth factor β and PPAR-γ (117). These findings were confirmed in two different experimental mouse models of colitis, either chemically (DSS)- or CD4-induced (118).

Other studies have focused their attention on the ω-6/ω-3 PUFA ratio. During the DSS-induced colitis model, mice administered with ALA-enriched diet, consequently resulting in a decreased uptake of LA, showed less severe colitis, with a markedly alleviated intestinal inflammation (119). The beneficial effects exerted by the ALA-enriched diet was probably due to the reduced PMN influx into the colonic mucosa, because of the decreased activity of both myeloperoxidase (MPO) and alkaline phosphatase. In addition, ALA supplementation blocked TNF-α and IL-1β up-regulation, by comparison with the control group.

Following studies were designed to use specific PUFA metabolites rather than precursors, with ω-3 EPA- and DHA-derived LMs as main candidates for both animal and clinical trials.

The first work involving ω-3 PUFA derivatives and IBD were conducted by using both TNBS- and DSS-induced colitis. Arita and colleagues demonstrated that RvE1 exerts protective effects in TNBS-induced intestinal inflammation, in terms of reduced body weight loss, colon shortening, and tissue damage, by reducing PMN flux into the colonic mucosa, and, at the same time, by limiting either the production of TNFα and IL-12, or the expression of pro-inflammatory enzymes, like COX-2. The authors also showed that the expression of the RvE1 receptor *ChemR23* was up-regulated in colonic mucosa of TNBS-treated animals (36).

Similar effects were observed in the DSS-induced model of colitis by Ishida et al., who demonstrated that repeated administrations of RvE1 were able to dampen colitis severity in terms of body weight loss, colon shortening, and histological score (41). Concomitantly, they observed a reduction in NF-kB phosphorylation, TNFα, IL-1β, and IL-6 levels in colonic tissues, along with higher levels of *ChemR23* mRNA, supporting a possible role for this receptor in the pathogenesis of intestinal inflammation (41). Other groups confirmed these findings additionally indicating that an interplay might exist between ALPI and RvE1 that ultimately leads to resolution of intestinal inflammation.

In 2011, Bento et al. showed that aspirin-triggered (AT)-RvD1 and RvD2 protect mice against both TNBS- and DSS-induced colitis (47). In this study, the preventive administration of these resolvins significantly ameliorated clinical manifestations, such as body weight loss, disease activity index, colonic damage, and colon shortening. Beside these clinical findings, they showed these mice to produce reduced levels of pro-inflammatory cytokines, and diminished activation of NF-kB pathway and expression of VCAM-1, ICAM-1, and leukocyte function-associated antigen-1. Finally, the authors demonstrated that blockage of LXA4 receptor (ALX), reversed the (AT)-RvD1protective effects in DSS-induced colitis, concluding that (AT)-RvD1 action may depend on ALX activation.

Other DHA-derived pro-resolving mediators, such as maresins, have also shown fundamental properties in experimental IBD. In fact, preventive or therapeutic administration of MaR1 (103) demonstrated for the first time that this DHA metabolite protects mice against both acute and chronic DSS-induced colitis, reducing disease activity index, colon shortening, body weight loss, and MPO activity. In addition, the authors demonstrated that MaR1 inhibited the production of pro-inflammatory cytokines like IL1-β, IL-6, TNF-α, and IFN-γ in colon tissue, together with down-regulation of NFk-B activation and diminished neutrophil transmigration in the inflamed mucosa (103). Similar results were obtained with the TNBS-induced model of colitis.

A very recent work from Gobbetti et al. shows that exogenous administration of LMs derived from ω-3 docosapentaenoic acid (ω-3 DPA), an intermediary product between EPA and DHA, named protectin D1*_n_*_−3 DPA_ (PD1*_n_*_−3 DPA_) and resolvin D5*_n_*_−3 DPA_ (RvD5*_n_*_−3 DPA_), was effective in preventing the acute model of DSS-induced colitis, in terms of reduced colon length, and microscopic damage score (120). These protective effects were partially linked to reduced granulocyte trafficking and PMN–endothelial interactions, which may occur downstream adhesion molecule activation. The translational impact of these data was determined not only by the ability of PD1*_n_*_−3 DPA_ and RvD5*_n_*_−3 DPA_ to reduce human neutrophil adhesion onto TNF-α-activated human endothelial monolayers, but also to the identification of ω-3 DPA metabolites in human colon biopsies. Using targeted LC-MS/MS-based LM metabololipidomics on colonic biopsies from controls and IBD patients they observed that LTB_4_, PGE_2_, and TX B_2_ were significantly increased in inflamed tissues in comparison with controls. Notably, they showed that RvD5*_n_*_−3 DPA_ and PD1*_n_*_−3 DPA_ were augmented in tissue biopsies from IBD patients compared with those from control. This finding on human IBD samples is in contrast with the fact that RvD5*_n_*_−3 DPA_ and PD1*_n_*_−3 DPA_ exert protective effects against chemically induced acute colitis, and warrants further investigation. There may be a dysfunctional susceptibility of cells targeted by these mediators in IBD. Moreover, it would be interesting to distinguish the effects of RvD5*_n_*_−3 DPA_ and PD1*_n_*_−3 DPA_ in patients with UC versus CD.

The last study that needs to be mentioned has been done by Meister and Ghosh, who treated IBD patient-derived biopsies with fish oil. They found reduced inflammation in terms of high IL-1a/IL-1b ratio in tissues derived from patients with UC, but not in tissues from patients with CD. These contrasting outcomes indicate that variations in diet composition may influence the success of a nutritional therapy for UC or CD patients (121). All mentioned animal studies are summarized in Table 1.

**Table 1:** Polyunsaturated fatty acid (PUFA) administration in animal models of IBD.

Study reference	Administered PUFA	Model of colitis	Outcomes
Viladomiu et al. (116)	CLA	DSS (pig)	Reduction of: DAI, TNFα, increase of: TGFβ and PPARγ
Bassaganya-Riera et al. (118)	CLA	DSS, CD4^+^ transfer (mouse)	Reduction of: inflammation, TNFα, increase of: TGFβ and PPARγ
Tyagi et al.(119)	Decreased LA/ALA ratio	DSS (rat)	Reduction of: DAI, intestinal inflammation, TNFα, and IL1β levels
Arita et al. (36)	RvE1	TNBS and DSS (mouse)	Reduction of: weight loss, colon shortening and tissue damaging, PMN infiltration, IL-12, TNFα, and COX-2
Ishida et al. (41)	RvE1	DSS (mouse)	Reduction of: weight loss, colon shortening and tissue damaging, NFk-B activation, TNFα, IL-1β, and IL-6
Bento et al. (47)	AT-RvD1, RvD2	TNBS and DSS (mouse)	Reduction of: weight loss, DAI, colon damage and shortening, pro-inflammatory cytokines, NFk-B activation, and adhesion molecules
Marcon et al. (103)	MaR1	DSS (mouse)	Reduction of: DAI, colon shortening, weight loss, myeloperoxidase activity, pro-inflammatory cytokines, NFk-B activation, and neutrophil transmigration
Gobbetti et al. (120)	PD1 and RvD5	DSS (mouse)	Reduction of: colon length and pro-inflammatory cytokines, leukocyte–endothelial interaction

It is worth of note that although the majority of pre-clinical studies on animal model of IBD are promising and provide strong or mild anti-inflammatory properties of ω-3 PUFAs (122–131), other works revealed that an abundant intake of dietary ω-3 PUFAs could even worsen the clinical signs of colitis (132–135). This discrepancy may be explained by different treatment and dose regimen, by different animal facility conditions, and different racemic mixture that could have been used to treat mice. In any case, this must be taken into consideration when animal studies need to be translated into clinical management of IBD.

## Clinical Application

As for animal models, many attempts have been done to prove ω-3 PUFA efficacy in human studies. The great therapeutic potential of ω-3 PUFAs has been also encouraged by some works reporting alterations in the production of pro-resolving LM. For example, Pearl and colleagues revealed the ω-6/ω-3 PUFA composition were altered in the inflamed gut mucosa of patients with active UC, in comparison with healthy samples (136). Additionally, Masoodi et al. reported that pro-inflammatory PUFA metabolites (PGD2, PGE2, TXB2, 5-HETE, 11-HETE, 12-HETE, and 15-HETE) not only were increased in the inflamed mucosa of patients with active UC, but their levels also correlated with the disease activity (137). Interestingly, our group recently characterized colonic biopsies isolated from patients with active UC showing that the production of pro-resolving DHA-derived metabolites was defective in inflamed mucosa in comparison with colon tissues from patients with UC in remission and healthy controls. This indicates that pro-resolving mechanisms are deficient in patients with active UC (20), suggesting that ω-3 PUFA administration can be exploited as a novel therapeutic approach to treat IBD.

The majority of studies that have been performed so far uses diet as way of delivery of ω-3 PUFAs, in combination or not with the conventional IBD therapies (138). John et al. found that the intake of dietary EPA and DHA was conversely correlated with the risk of developing incident UC (139). Similarly, in a cohort of patients with CD, the dietary DHA intake was conversely correlated with the development of incident CD, with statistical significance (140). Moreover, clinical trial for CD and UC revealed the beneficial effects of ω-3 enriched diet (141–151) in terms of clinical and histological parameters. Among these, Belluzzi et al. showed that in patients with CD in remission, fish-oil enriched diet is effective in decreasing relapse frequency (146). In another multicenter, randomized, double-blind, clinical trial the beneficial role of fish-oil administration in patients with UC was demonstrated. The positive clinical outcome was expressed in terms of reduced rectal leukotriene B4 (LTB4) levels, improvements in histological scores, and gain of weight.

Omega-3 PUFA administration may also be effective in pediatric patients. In children with CD treated with mesalazine, diet supplementation with ω-3 PUFAs significantly reduced the frequency of relapse within 1-year observation in comparison with patients receiving placebo, consisting in olive oil (145).

However, in a clinical trial (EPIC-1 and -2) conducted by Feagan et al. the efficacy of a mixture of ω-3 PUFA was revised; in fact, the treatment was not effective in preventing relapse and maintaining remission in CD patients (152). All clinical studies are summarized in Table 2.

**Table 2:** Clinical studies with the use of polyunsaturated fatty acids (PUFAs) in inflammatory bowel disease.

Study reference	Treatment	Disease	Outcome
Romano et al. (145)	ω-3	CD	Lower relapse than placebo
Belluzzi et al. (146)	ω-3	CD	Maintenance of remission compared with placebo
Feagan et al. (152)	ω-3	CD	No effects
Stenson et al. (142)	ω-3	UC	No changes compared with placebo
Barbosa et al. (141)	ω-3	UC	Decreased oxidative stress compared with placebo
Lorenz-Meyer et al. (157)	ω-3 and low carbohydrate diet	CD	No amelioration compared with placebo
Nielsen et al. (158)	ω-3 and ω-6, arginine and ribonucleic acids, and prednisolone	CD	No significative reduction of Crohn’s disease activity index (CDAI) compared with placebo
Geerling et al. (147)	ω-3 and antioxidant	CD	Increase of antioxidants; better resolving PUFA profiles in treated compared with placebo
Nielsen et al. (148)	ω-3	CD	Reduced pro-inflammatory cytokines and CDAI
Eivindson et al. (159)	ω-3 and corticosteroids	CD	No difference between groups
Brunborg et al. (149)	ω-3	UC/CD	Reduced joint pain
Bjørkkjaer et al. (150)	ω-3	UC/CD	Reduced disease activity compared with placebo
Seidner et al. (151)	ω-3, fiber, and antioxidant	UC	Reduced use of prednisone compared with placebo
Salomon et al. (143)	ω-3	UC	Improvement in seven patients

The opportunity of clinical application for PUFAs has been evaluated by few systematic reviews and meta-analyses. For example, the study by Turner and colleagues found significant positive effects of ω-3 PUFA supplementation in CD patients. However, these conclusions derived from only six trials that are highly heterogeneous. Analysis of three clinical trials on ω-3 PUFA administration in patients with UC described no significant outcome. Thus, the authors concluded that data available were insufficient to prescribe the use of ω-3 PUFAs for the maintenance of remission in CD and UC (153, 154).

Overall, the studies conducted so far are elusive and displayed no real evidence of efficacy (138, 155–159). This might be due to different reasons: (a) the ω-3-based diet needs to be tightly controlled in IBD patients; (b) the administration of ω-3 PUFA (DHA or EPA) through diet is not effective because of insufficient intestinal absorption due to ulcers or because of biochemical modification of PUFAs when they are in the systemic circulation; (c) EPA and DHA are general precursors of a plethora of specific pro-resolving lipids, that, by definition, are locally and timely regulated. Therefore, the administration through the diet does not help to finely control such metabolism; (d) patients may harbor genetic predisposition impeding the correct DHA or EPA metabolism, thus leading to insufficient production of bioactive pro-resolving LMs.

## Concluding Remarks

In IBD patients, diet and lifestyle changes, conventional or newly identified drugs, do not always resolve inflammation and relieve symptoms of the disease. One of theories formulated in the last few years is that anti-inflammatory agents do not alter the course of the disease, because naturally occurring resolution programs may have been subverted. Few studies, including findings from our group, showed that eventual dysfunctions in resolution pathways and/or deficits in precursors of pro-resolving mediators, such as ω-3 PUFAs, may lead to persistent inflammation and provoke alteration in gut mucosa homeostasis, thus being part of IBD pathogenesis. For this reason, the use of pro-resolving PUFAs, particularly the ω-3 ones, brings new possibilities to the treatment of IBD, and could be of great interest to pharmacological industry.

Although numerous pre-clinical and clinical studies employing the use of PUFAs, either as fatty acid precursors or single metabolites, showed controversial results, there is still much more to discover about the beneficial effects of these molecules, particularly in the IBD field. It would be important not only to uncover new cellular and molecular processes modulated by PUFAs under gut inflammatory conditions, but also to unveil novel biosynthetic pathways of these pro-resolving LMs that may likely be dysregulated in IBD. Ways of delivery, safety, dosage, and regimen treatment, and interaction with other drugs should also be further addressed in order to establish the most efficient replacement therapy. We suppose that either PUFAs or new medications specifically promoting resolution pathways will be much better tolerated by patients with IBD, mimicking the physiological processes through which inflammation naturally occurs in the organism, with the advantage of avoiding immune suppression.

## Author Contributions

FU, FR, SD, and SDA conceived and wrote the manuscript, and realized the figure.

## Conflict of Interest Statement

The authors declare that the research was conducted in the absence of any commercial or financial relationships that could be construed as a potential conflict of interest.
